# The Impact of Coercive Measures on the Therapeutic Relationship Between Patients and Nurses in the Acute Psychiatric Care. An Integrative Review

**DOI:** 10.1111/jpm.70012

**Published:** 2025-07-15

**Authors:** Florian Wostry, Sabine Hahn, Berta Schrems

**Affiliations:** ^1^ University of Vienna Vienna Austria; ^2^ School of Nursing Bern University of Applied Sciences Switzerland

**Keywords:** coercion, dehumanisation, nurse patient relationship, psychiatric nursing, psychiatry

## Abstract

**Introduction:**

The reduction of coercion requires a stable therapeutic relationship. It is generally assumed that coercive measures have a negative effect on the therapeutic relationship, but little is known about the specific impact.

**Question:**

What is the impact of coercive measures in acute psychiatric care on the therapeutic relationship between nurses and patients?

**Method:**

An integrative review and a thematic analysis were undertaken.

**Results:**

Theme 1, labelled ‘Destructive effects’, encompasses three subthemes: ‘Loss of trust’, ‘Power imbalance’ and ‘Engagement reduction’ and highlights the negative impact on the therapeutic relationship. Theme 2, entitled ‘Nursing dilemma’, with the subtheme ‘Dehumanisation’ discusses the inherent conflicts faced by mental health nurses. Theme 3, ‘Reinforcement’, suggests potential enhancements in therapeutic relationships.

**Discussion:**

Key characteristics of the therapeutic relationship, such as providing support, meeting at eye level, empathy and trust, can suffer damage from coercive measures and diminish a fundamental aspect of psychiatric nursing. Moreover, the absence of a therapeutic relationship can foster behaviours that prompt additional coercive measures, creating a negative cycle with adverse effects for all involved.

**Implications for Practice:**

Nurses need to be aware of the effects of coercive measures on therapeutic relationships and use coercive measures as a last resort.

**Summary**

- What is known on the subject?
  ○ A strong therapeutic relationship is essential for recognising changes in the patient's behaviour and mood that could potentially lead to violence or aggression at an early stage. The therapeutic relationship is therefore a central element of de‐escalation and is vital for curbing coercive measures.
  ○ The use of coercive measures is a physical and psychological challenge for patients and nursing staff. Patients in particular report traumatic experiences in this regard.
- What the paper adds to existing knowledge?
  ○ Coercive measures negatively affect the therapeutic relationship between patients and nurses and thus impair engagement.
  ○ Coercive measures create a nursing dilemma in which nurses deliberately dehumanise themselves, which is counterproductive for the key characteristic of the therapeutic relationship.
  ○ In some cases, nurses report beneficial effects on the therapeutic relationship.
- What are the implications for practice?
  ○ When applying coercive measures, nurses should consider this negative impact on the therapeutic relationship. Nursing staff should critically question coercive measures and develop sustainable patient‐centred, recovery‐oriented alternatives to strengthen trust and cooperation between patients and nursing staff and achieve more effective treatment outcomes in the long‐term.
  ○ The establishment and maintenance of a stable therapeutic relationship must play a central role in psychiatric care, as all of its characteristics are an important protective factor against dehumanisation and the use of coercive measures.
  ○ Future research on the effects of coercive measures on therapeutic relationships should take into account the longitudinal process of the use of these measures.

## 1 Introduction

Coercive practices have long been a component of psychiatric acute care and remain controversial, as their use is not consistent with human rights‐based mental health care (Sashidharan et al. 2019). Coercion is a broad concept that includes both informal and formal practices. Informal coercion may involve subtle or overt communication, manipulation, misuse of power or enforcing conformity to rules and norms (Beeri et al. 2025; Paradis‐Gagné et al. 2021). This paper focuses on formal physical coercive measures. The European Committee for the Prevention of Torture and Inhuman or Degrading Treatment or Punishment [CPT] emphasises that coercive measures should only be used to protect patients from self‐harm and to prevent endangering others, whereby the principles of legality, necessity, proportionality and responsibility must be observed. Following formal types of coercive measures that are widely used in psychiatric acute care are reported: seclusion, physical restraint, mechanical restraint and chemical restraint. Seclusion is the involuntary placement of a patient in a locked room. This seclusion room should be specially designed to ensure the patient's safety and provide a calming environment. Physical restraint, also known as holding, involves staff members using physical force to hold or immobilise a patient. This may include restraining movements or actions by direct physical intervention. The use of specific equipment or devices, such as straps, to immobilise is called mechanical restraint. Chemical restraint involves the involuntary administration of drugs (oral, intramuscular, intravenous and nasal) to control a patient's behaviour. This practice aims to sedate or tranquillise the patient, typically to manage agitation or disruptive conduct (CPT 2017). Such forced medication is usually administered as part of a holding or mechanical restraint, as it is only by restricting movement that the medication can be administered. It should be noted that in scientific literature, restraint often refers to physical restraint, although physical restraint is also used as a generic term for mechanical restraint and/or manual restraint in the form of physical grabbing (Negroni 2017), the same as holding (CPT 2017). Mechanical restraint in psychiatry is closely related to manual restraint, as the latter is typically used to initiate the former. However, manual restraint can also be used independently without leading to mechanical restraint (Negroni 2017). In the following, we will use the term coercive measures for formal types of coercive measures to emphasise the aspect of forced use.

A systematic review (Chieze et al. 2019) indicates that coercive measures may result in adverse physical or psychological effects. The incidence of post‐traumatic stress disorder following such interventions is estimated to be between 25% and 47%. Additionally, it is also noted that subjective perceptions vary widely between individuals and are predominantly associated with negative emotions. Chieze et al. (2021) emphasise that coercion continues to be used despite being known to cause harm. Acting against ethical and professional values can also lead to ambivalence and cognitive dissonance among nursing staff (Paradis‐Gagné et al. 2021). The ethical justification of coercion is controversial; while some defend its use in rare emergencies, others advocate its abolition due to violations of fundamental rights. This highlights the ethical responsibility of professional nursing associations to promote alternatives, encourage critical reflection, and advocate for systemic change. As Lorem et al. (2015) state, good clinical practice cannot be separated from the formal moral evaluation of coercion. The CPT (2017) states that coercive measures should only be used as a last resort, after all other options to prevent someone from harming themselves or others have been exhausted, and for the shortest time possible. Once the danger has passed, the person should be released immediately.

The European Union funded network Fostering and Strengthening Approaches to Reducing Coercion in European Mental Health Services [FOSTREN] emphasises that as long as coercion is used in psychiatry, it is essential to explore its effects (FOSTREN 2022). The World Health Organisation [WHO] (2021), with its Comprehensive Mental Health Action Plan 2013–2030, and the United Nations (2017), with its 2030 Agenda for Sustainable Development, are currently strengthening research on coercive measures. The objectives have been chosen in such a way that human rights in psychiatry will be ensured and the dignity of people will be safeguarded through the gradual abolition of coercive measures.

Complex interventions such as Safewards (Bowers et al. 2015) which include de‐escalation, appear to be effective in reducing coercive measures (Baumgardt et al. 2019). Therefore, it is important to promote successful de‐escalation strategies. A prerequisite for successful de‐escalation is a strong therapeutic relationship [TR] based on trust, fairness, consistency, and mindfulness (Goodman et al. 2020). Such a TR makes it possible to recognise changes in the patient's behaviour and mood that could lead to violence or aggression (National Institute for Health and Care Excellence 2015). In addition, a good TR allows for in‐depth knowledge of the patient to be gained in order to better assess their reactions in acute situations (Hartley et al. 2020; McAndrew et al. 2014; Hopkins et al. 2009). According to McAndrew et al. (2014), acute psychiatric situations require the creation of a therapeutic space that is free from negative influences and filled with meaningful interactions between nurses and patients. This therapeutic space is at risk of being damaged by coercive measures (Gilburt et al. 2008) as well as the TR is at risk of being disrupted by coercion (Theodoridou et al. 2012). In the literature, the TR is frequently mentioned and described in the context of psychiatry. However, there are different definitions of TR. In this paper, a broad definition of TR is used.

The American Psychiatric Nursing Association [APNA] emphasises that psychiatric nursing is characterised by close TR (APNA 2022). The TR is defined as an interaction between the nurse and the patient in which all parties contribute to a climate of a positive focus on health promotion (Townsend 2011). The TR is underpinned by a person‐centred, values‐based approach to care that emphasises personal connections and consistent care (National Institute for Health and Care Excellence 2015). In inpatient psychiatric wards, the therapeutic role of nurses is essential as they have the most time‐intensive contact with patients. The TR promotes patient recovery and is considered a key factor in treatment success (McAndrew et al. 2014; Hopkins et al. 2009). In this regard, Shattell et al. (2007) point out that patients want a TR that is characterised by sufficient time, understanding and competent care. Listening to and responding to patients' needs is crucial if the TR is to reach its full potential. In their literature review, Dziopa and Ahern (2008) identified the following nine characteristics that a nurse needs for a successful TR: empathy and understanding, individuality, support, being there, being genuine, meeting at eye level, respect, setting boundaries and being assertive. According to Hartley et al. (2020), establishing and maintaining a TR is complicated by the lack of clear clinical recommendations. There is evidence suggesting that the TR is more negatively rated the more intense coercion is experienced (Theodoridou et al. 2012). However, little is known about the specific effects of these measures on the TR. Yet, awareness of the harmful impacts of coercive measures can only be created if it is clear how they affect the TR. This integrative review addresses this gap by synthesising existing evidence and is, to our knowledge, the first to explore this interplay from both patient and nurse perspectives.

## 2 Aim

The aim of this review is to systematically examine what is known about the effects of coercive measures on the TR between patients and nursing staff in acute psychiatric care. The research question is: What is the impact of coercive measures in acute psychiatric care on the TR between nurses and patients? The results will be used to identify measures that can be taken to improve the TR in critical situations.

## 3 Method

To develop a holistic picture of the impact of coercive measures on the TR between patients and nurses in acute psychiatric care, an integrative review was conducted. This review followed the five stages described by Whittemore and Knafl (2005): problem identification (see background and aim), literature search, data evaluation, data analysis, and presentation. To increase the usefulness of a systematic review for its users, authors are encouraged to provide a transparent account of the review process and results (Page et al. 2021). To ensure these quality features, the PRISMA Checklist 2020 was used for this review, and the protocol was registered in PROSPERO (CRD42024541642).

### 3.1 Literature Research

The initial literature search began in June 2023, following the specification of search strings. The final literature search was completed in February 2024, without a time limit and restricted to English and German literature in the following databases: Cochrane Library (via Cochrane Library), Psyndex (via PubPsych), Medline (via PubMed), Cinahl (via EBSCO‐host), APA psyc Info (via EBSCO‐host), Soc Index (via EBSCO‐host) and Web of Science Core Collection (via Web of Science). The search terms and Boolean operators used are listed in Table 1.

**TABLE 1:** Search terms and Boolean operators.

**In English**						
(‘therapeutic relationship’ OR ‘helping relationship’ OR ‘nurse–patient relationship’ OR ‘trusting relationship’ OR ‘therapeutic alliance’)	AND	(‘coercive measure*’ OR ‘involuntary treatment’ OR ‘compulsory treatment’ OR ‘mechanical restraint*’ OR belt* OR ‘physical restraint*’ OR holding OR restraint* OR ‘isolation room*’ OR seclusion* OR ‘restrictive intervention*’ OR ‘forced medic*’ OR ‘emergency sedation’ OR ‘compulsory medic*’ OR ‘medical restraint*’ OR ‘chemical restraint*’)	AND	psychia*	AND	nursing
**In German**						
(‘therapeutische Beziehung’ OR ‘Pflegebeziehung’ OR ‘Pflegeperson‐Patienten Beziehung ‘OR’ Pflegefachperson‐Patienten Beziehung’ OR ‘therapeutische Allianz’)	AND	(Zwangsmaßnahme* OR ‘unfreiwillige Behandlung’ OR ‘freiheitsentziehenden Maßnahme*’ OR Zwangsbehandlung OR ‘mechanische Fixierung’ OR Gurtfixierung OR Schutzfixierung OR Fixierung OR ‘physische Fixierung’ OR festhalten OR Isolierzimmer OR Isolationszimmer OR Isolierung OR Isolation)	AND	psychia*	AND	Pflege

In addition, the berry‐picking method of searching reference lists for appropriate inclusion criteria was used. To facilitate the search strategy, two researchers with experience in literature research were consulted. In the research question, the term coercive measures includes all measures by nurses such as mechanical restraint, physical restraint, seclusion and forced medication. Legal conditions over which nurses have no control are not included, such as involuntary treatment orders or the requirement that the patient is not allowed to leave the ward in the form of an involuntary admission. The research question is based on Murdoch University's PICo strategy (2024). The inclusion and exclusion criteria for the literature search are listed in Table 2.

**TABLE 2:** Inclusion and exclusion criteria.

	Inclusion criteria	Exclusion criteria
Population	– Nurses working in the psychiatric setting and who used coercive measures OR – Psychiatric patients in the psychiatric setting who experienced coercive measures	– Nurses working in other settings than psychiatry or mental health OR – Non‐psychiatric Patients. Psychiatric and non‐psychiatric patients in other health care settings, like oncology or surgery etc.
Interest	– When there is a reference to the TR	– When there is no reference to the TR
Context	– Coercive measures in psychiatry: mechanical restraint, physical restraint, seclusion and forced medication	– Involuntary treatment orders – Involuntary admission
Type of study	– No limitations	– None scientific literature (grey literature, project and praxis reports)
Language	– English & German	– Other languages
Year	– No limitations	– No limitations

**FIGURE 1:**
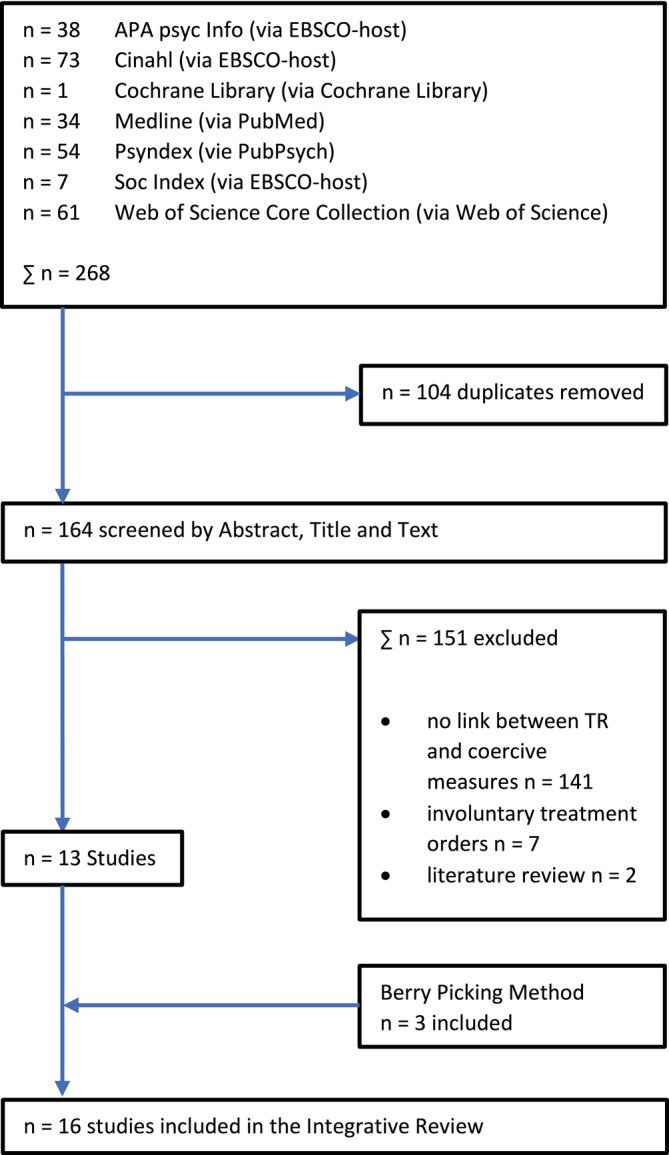
Literature research.

The literature search conducted by the first author resulted in a total of 268 studies. After removing duplicates, 164 studies remained. Abstract, title and text were then screened using the inclusion and exclusion criteria. If systematic reviews were found, the respective original study was used, which either appeared as an individual study in the literature search anyway or by means of berry picking. Three studies were added using the berry picking method, resulting in a total of 16 included studies. The search did not reveal any none scientific literature like grey literature project and praxis reports. All included studies are in English language and have a qualitative research design. The process of the literature search can be seen in Figure 1.

### 3.2 Data Evaluation

The third stage of the integrative review is to assess the quality of the selected publications. Because all included studies have a qualitative design, they were assessed for scientific quality criteria using the Critical Appraisal Checklist for Qualitative Research (The Joanna Briggs Institute 2017). The eligibility of the studies was assessed by all three authors. No automation tools were used in that process. In all studies, researchers, participants, and the environment are adequately described. All 16 studies were approved by an ethics committee. The analysis of the data collection is described in each study. All sources used by the authors are clearly listed. The selected studies were positively evaluated and therefore included in the integrative review. At the end of the quality appraisal, the 16 studies were confirmed and reviewed by all three authors.

### 3.3 Data Analysis

Braun and Clarke's (2006) six‐step content analysis method was used to analyse the content of the studies. First, the study results and discussion part were read thoroughly to gain familiarity. Initial codes were then generated by identifying key terms or phrases. These codes were then mapped into broader themes or patterns. The researchers then reviewed these themes to ensure that the data were accurately represented. Once this was confirmed, the themes were defined and labelled. Finally, the researchers interpreted the findings and analysed the significance of the identified themes in relation to the research question. Disagreements were discussed until a consensus acceptable to all three researchers was reached.

### 3.4 Ethical Considerations

Ethical clearance is not required as this integrative review is based on published literature.

## 4 Results

The range of research designs of the sixteen identified studies includes phenomenological approaches, qualitative content and inductive analysis, ethnographic and realist strategies, in‐depth interviews and descriptive techniques, representing a broad spectrum of qualitative research methods. Of these studies, seven were from the United Kingdom, three from Spain, two from Canada and one each from China, Iran, Ireland and New Zealand. Two studies (Duffy et al. 2024; Knowles et al. 2015) are based on a research question aimed at investigating the impact of physical restraint on the TR. The remaining studies did not include a specific research question focusing on the impact of coercive measures on the TR, but the findings do show consequences in this regard (Bigwood and Crowe 2008; Chambers et al. 2015; Fereidooni Moghadam et al. 2014; Jacob et al. 2019; Kodua and Eboh 2023; Li et al. 2023; Ling et al. 2015; Manzano‐Bort et al. 2021; McKeown et al. 2020; Moreno‐Poyato et al. 2021; Moyles et al. 2023; Pérez‐Toribio et al. 2022; Tulloch et al. 2022; Wilson et al. 2017). Table 3 provides an overview of these studies and their findings on the consequences of coercive measures on TR, listing author, year, country, research design, population, type of coercive measure and findings. Regarding the type of coercive measures, the studies by Chambers et al. (2015), Ling et al. (2015) and McKeown et al. (2020) only used the term restraint without defining exactly which restraint measures were involved. Fereidooni Moghadam et al. (2014) discuss physical restraint and note that this includes limb holders, safety vests and bandages, which also implies mechanical restraint. Li et al. (2023) write about physical restraint, and it becomes apparent from the interview results that this pertains to belts indicative of mechanical restraint.

**TABLE 3:** Study overview.

Authors, year, country	Research design	Facility, population	Type of coercive measure	Knowledge gained
Bigwood and Crowe (2008) New Zealand	– Phenomenology	– Acute adult inpatient service – *n* = 7 registered comprehensive nurses (registered general and psychiatric) or registered psychiatric nurses	– Physical restraint	– Can form a bond – Conflict with therapeutic role
Chambers et al. (2015) England	– Qualitative inductive analysis processes	– Three adult acute psychiatric care units – *n* = 12 registered mental health nurses	– Restraint [not specified if seclusion, chemical, physical or mechanical restraint]	– Could destroy and ruin the relationship – Creates personal and professional conflict
Duffy et al. (2024) United Kingdom	– An interpretative phenomenology analysis	– Secure hospital which provides low and medium secure care for adults – *n* = 5 service user – *n* = 3 qualified nurses – *n* = 2 two health‐care workers	– Physical restraint	Service users' perspective: – Distress and disempowerment – Breakdown in the relationship and loss of trust – Reevaluation of the TR – TR remained unchanged – Disengagement from the TR – Staffs' perspective: – Balancing professional roles and responsibilities within the relationship
Fereidooni Moghadam et al. (2014) Iran	– Qualitative content‐analysis	– Psychiatric hospitals *n* = 14 nurses	– Physical restraint[includes devices designed to limit a patient's physical movements such as limb holders, safety vests and bandages]	– Damage to the relationship – Relationship will be better
Jacob et al. (2019) Canada	– Interpretative phenomenological analysis	– Inpatient psychiatric unit – *n* = 19 patients – *n* = 21 nurses	– Mechanical restraint	Patients' perspective: – Resoundingly unsettling for the nurse–patient relationship – Apprehensive over the TR – Nurses' perspective: – Can affect the nurse–patient relationship – Distancing from physical work to converse nurse–patient relationship
Knowles et al. (2015) England	– In‐depth qualitative interviews	– Medium secure service – *n* = 8 forensic patients	– Physical restraint [no detailed description available]	– Power imbalance in the staff–patient relationship – Sadistic, feeling vulnerable, fearful and distrusting of staff – Abusive, degrading, traumatic experience
Kodua and Eboh (2023) England	– Descriptive phenomenological study	– Four inpatient adolescent mental health hospitals – *n* = 12 registered mental health nurses – *n* = 4 nursing assistantsn = 3 senior nursing assistants	– Physical restraint	– Distressing for all – It does not really damage the TR – Damage to relationship is only temporary – Strengthens the relationships – Long‐term damage is rare
Li et al. (2023) China	– A descriptive approach to the semi‐structured interview	– Public psychiatric hotspital – *n* = 26 patients	– Physical restraint [with belts]	– Deteriorated the nurse–patient relationship – Loss of mutual understanding and trust
Ling et al. (2015) Canada	– Qualitative Analysis	– Centre for addiction and mental health – *n* = 55 postrestraint event inpatient debrief forms filed in by inpatients	– Restraint [not specified if seclusion, chemical, physical or mechanical restraint]	– Damaged relations with staff – Losing trust and negative emotions (trauma, anger, resentment, loneliness and sadness) – Small number of inpatients reported no effects
Manzano‐Bort et al. (2021) Spain	– A qualitative study design with a phenomenological‐hermeneutic approach	– Six short‐ and medium‐stay inpatient units of a specialised mental health care network – *n* = 35 mental health nurses	– Mechanical restraint	– It's not therapeutic – Feelings: stressful, unpleasant, exhausting, frustration, impotence, anger and even sadness, pity, guilt and disappointment – User shun the nurse – Distancing form the patient
McKeown et al. (2020) England	– Qualitative: rapid ethnography observations and semi‐structured interviews	– 14 acute mental health wards within seven national health service trusts – *n* = 32 service users – *n* = 130 staff (the majority of the staff comprised registered mental health nurses and healthcare assistants)	– Restraint [not specified if seclusion, chemical, physical or mechanical restraint]	Service users' perspective: – Individual and collective traumatic impacts upon therapeutic relations – Being distressed – Intensivates powerlessness, aggression and violence
Moreno‐Poyato et al. (2021) Spain	– A qualitative descriptive study design	– Former adult patients without disclosure of the facility – *n* = 11 service users	– Mechanical restraint – Pharmacological restraint – Seclusion	– TR can be easily broken – Difficult to establish a TR – A key measure of the TR is the absence of chemical and mechanical containment
Moyles et al. (2023) Ireland	– A qualitative study	– Two units in one forensic mental health service – *n* = 10 registered forensic mental health nurses	– Physical restraint	– Impact on the TR – Power imbalance – Loss of trust – Emotional impact for both groups – Patients becoming more withdrawn, hesitant and less likely to engage therapeutically following physical restraint – Hinders patient from participating therapeutic work – Viewing staff as more authoritarian – Rebuilding the TR needs time
Pérez‐Toribio et al. (2022) Spain	– Qualitative descriptive design	– Acute psychiatric admission ward – *n* = 10 mental health nurses	– Mechanical restraint	– Jeopardise TR – Impacted on the caring aspect of the mental health nurse role – Countertherapeutic desensitisation strategies – Broken trust – Distances the patient from health personnel – Will never strengthen the therapeutic bond
Tulloch et al. (2022) United Kingdom	– Qualitative interview study	– High‐security forensic service for male patients – *n* = 7 registered nurses – *n* = 5 non‐registered nurses	– Seclusion	– Nurse–patient relationship is not seen – Difficult to maintain a TR – Frustration
Wilson et al. (2017) United Kingdom	– Realist epistemological framework	– Variety of adult psychiatric wards – *n* = 13 patients – *n* = 6 nurses (ranging from student nurses to senior nurses with supervisory responsibilities) – *n* = 16 other staff	– Physical – Chemical restraint	Patients' and staffs' perspective: – Negative impact on patient–staff relationships – Mainly described as damaging the staff–patient relationship – Loss of trust – Short‐term damage Patients' perspective: – Long‐term damage – Makes relationship better – No impact on the TR

### 4.1 Generated Themes

Using Braun and Clarke's (2006) method of content analysis, a total of three themes were generated: ‘Destructive effect’, ‘Nursing dilemma’ and ‘Reinforcement’. The ‘Destructive effect’ theme consists of the three subthemes ‘Loss of trust’, ‘Power imbalance’ and ‘Engagement reduction’. The ‘Nursing dilemma’ theme includes the subtheme ‘Dehumanisation’.

#### 4.1.1 Theme 1: Destructive Effect

With the exception of Bigwood and Crowe (2008), all of the included studies indicate destructive effects of coercive measures on the TR between patients and nurses, ranging from mild to severe. Specific details are not provided in the studies. Pérez‐Toribio et al. (2022) and Jacob et al. (2019) highlight jeopardised and unsettling effects on the TR. Others, such as Duffy et al. (2024) and Li et al. (2023), note a deterioration and breakdown of the TR. Ling et al. (2015) mention damage to the TR due to negative experiences of coercion. Moreno‐Poyato et al. (2021) emphasise that the TR can easily break down due to coercive measures, and Wilson et al. (2017) point out that the TR is always damaged. Chambers et al. (2015) dramatically state that coercive measures ruin the TR. Additionally, Tulloch et al. (2022) report that the TR becomes invisible to aggressive patients.

It's not a comfortable thing to do to the patient or yourself because it ruins the relationship. [nurse] (Chambers et al. 2015, 292)

It's always gonna damage the therapeutic relationship …. [staff] (Wilson et al. 2017, 505)

The impact of coercive measures on the TR can be profound and lead to loss of trust and power imbalances. Despite variations in the duration of these negative effects, both patients and nurses report significant short‐ and long‐term consequences, highlighting the profound challenges faced in maintaining the TR following such measures (Kodua and Eboh 2023; Moyles et al. 2023; Wilson et al. 2017).

McKeown et al. (2020) report traumatic effects at both individual and collective levels. Individuals were distressed by being subjected to coercion and staff were also upset by these incidents. Patients' expressions of aggression and violence were often preceded by feelings of powerlessness or not being listened to, which were then exacerbated by the experience of restraint and forced medication. Coercive measures not only deteriorate and damage the TR (Li et al. 2023; Ling et al. 2015), but also hinder their development (Moreno‐Poyato et al. 2021). Reports from service users suggest that the negative emotions resulting from the restraint may be contingent on the TR (Duffy et al. 2024). The duration of the negative effects on the TR varies, with both patients and carers reporting short‐ and long‐term effects.

The subthemes of ‘Loss of trust’, ‘Power imbalance’ and ‘Engagement reduction’ provide a more detailed overview of the destructive effects on the TR.

##### 4.1.1.1 Subtheme: Loss of Trust

In particular, trust, an important key element of the TR, is compromised by coercive measures. This is confirmed by both patients and nurses (Knowles et al. 2015; Li et al. 2023; Ling et al. 2015; Manzano‐Bort et al. 2021; Moyles et al. 2023; Pérez‐Toribio et al. 2022; Wilson et al. 2017).

In some cases, the patient may reject the nurse due to feelings of anger (Manzano‐Bort et al. 2021), treatment (Wilson et al. 2017) or pain (Li et al. 2023). Conversely, the nurse may distance themselves from the patient, particularly if the psychomotor agitation of the patient has been intense and violent (Manzano‐Bort et al. 2021).

I do not trust Nurse A and Nurse B, because they brought me the pain last time. [patient] (Li et al. 2023, 1776)

It left me with … a total distrust … a total vote of no confidence and no faith in anything they did, wanting to have absolutely nothing to do with any of them … all the time this is in my mind how they've treated me and how they treat other people, and obviously that affects relationships with them. [patient] (Wilson et al. 2017, 504)

##### 4.1.1.2 Subtheme: Power Imbalance

Coercive measures in mental health care create or reinforce a power imbalance within the TR and the overall therapeutic process. Patients often feel powerless and unheard during restraint, exacerbating feelings of vulnerability and distrust towards nurses, leading to traumatic experiences and perceptions of abuse of power within the system.

Although coercive measures may be justified for safety reasons in mental health care, they inevitably lead to a power imbalance that permeates the TR (Moyles et al. 2023). This power imbalance can hinder the development of trust and mutual respect, impede the therapeutic process, and potentially lead to further conflict and distress. The experience of being restrained exacerbated feelings of powerlessness or not being heard by patients (Duffy et al. 2024; McKeown et al. 2020). Patients report too much solidarity among nurses, so that in some situations, decisions are made to the detriment of the patient's perspective (Knowles et al. 2015). Patients perceive it as an abuse of power and feel vulnerable when nurses behave sadistically or use restraints as a means of control and punishment (Duffy et al. 2024; Knowles et al. 2015; Li et al. 2023).

It has been traumatic. I feel a victim of a corrupt system … [patient] (Ling et al. 2015, 389)

##### 4.1.1.3 Subtheme: Engagement Reduction

Coercive measures reduce patients' adherence to treatment (Li et al. 2023) and increase their withdrawal and reluctance. This leads to reduced engagement in therapeutic work (Moyles et al. 2023). Patients disengaged themselves from the nurses, which was related to the upcoming fear after coercive measures that made it impossible to seek communication, support, and care from nurses. Additionally, nurses experience assaults, threats, and verbal abuse, leading to emotional impacts that hinder their therapeutic effectiveness, which is also a disengagement from the TR (Duffy et al. 2024).

I do not always trust them (nurses), because they treated me like an animal. They only seemed to care about they [sic] work. I would like to refuse the medication because I do not trust them. [patient] (Li et al. 2023, 1775–1776)

#### 4.1.2 Theme 2: Nursing Dilemma

The use of coercive measures poses nurses with inherent conflicts juxtaposed to their TR responsibilities, potentially resulting in frustration and counterproductive dehumanisation. According to Bigwood and Crowe (2008), nursing staff are often torn between their therapeutic role and the culture of control that prevails on the ward. These conflicts can lead to personal and professional dilemmas. Chambers et al. (2015) emphasise that nurses are concerned about the potential for coercive measures to compromise the TR. The possibility of damaging the TR is cited as one of the main concerns of nurses. Duffy et al. (2024) highlight that staff are assigned conflicting roles and responsibilities within TR. This becomes particularly evident in the use of coercive measures.

You do need to build that basic relationship with someone, but how can you do that when you've got to constantly restrain them. They're not going to accept you. [staff] (Duffy et al. 2024, 136)

Manzano‐Bort et al. (2021) and Tulloch et al. (2022) illustrate the emotional toll that nurses have to pay when using restraint. Nurses perceive this task as aggressive and unpleasant and feel uncomfortable, even though it is only used as a last resort. Participation in coercive measures can also lead to moral injury, as described by Pérez‐Toribio et al. (2022). These injuries not only remain at a personal level, but also hinder the nurses to carry out their nursing duties effectively.

It's never great to restrain a patient […] But we are demonising mechanical restraints; I wonder. I wonder whether we are demonising them because they are not therapeutic. [nurse] (Manzano‐Bort et al. 2021, 8)

The subtheme of dehumanisation has also emerged in the ‘nursing dilemma’:

##### 4.1.2.1 Subtheme: Dehumanisation

Pérez‐Toribio et al. (2022) found that nurses deliberately desensitise themselves antitherapeutic to protect themselves from moral injury during coercive measures in order to avoid empathising with the patient. According to Duffy et al. (2024), the nurses are attributed with appearing empty, detached and showing no emotion. Furthermore, the nurses are accused of using disrespectful language (Knowles et al. 2015).

… you do what you have to do and it's over, you desensitize to this kind of thing … I have been desensitizing myself, […] [nurse] (Pérez‐Toribio et al. 2022, 693)

He came across as blank. Like he'd detached himself from it. Like he had no emotions. [patient] (Duffy et al. 2024, 131)

#### 4.1.3 Theme 3: Reinforcement

Compared to the many negative effects, there are only a few studies suggesting a strengthening of the TR. It is striking that this is only reported from a nurse's perspective. Reported amplifications include improvements, strengthening, and increased bonding within the TR.

In the study by Fereidooni Moghadam et al. (2014), nurses observed an improvement in the pre‐established TR following the calming of patients during coercive measures. Similarly, findings from Bigwood and Crowe (2008) and Kodua and Eboh (2023) highlighted positive effects on the nurse–patient relationship. In the study of Kodua and Eboh (2023) four nurses reported that manual restraint strengthened their relationships with young people, using terms such as ‘improves’ and ‘strengthens’. Despite some uncertainty about this effect, two highlighted the opportunity restraint provided to connect with young people at vulnerable times, fostering closer relationships.

And they actually have the effect in some cases of forming a bond between the patient and the staff member. [nurse] (Bigwood and Crowe 2008, 219)

## 5 Discussion

This integrative review examined and summarised the current evidence on the impact of coercive measures on the TR between patients and nurses. All included studies have a qualitative design. Three key themes emerged from the analysis. Theme 1, ‘Destructive effects’, includes the subthemes ‘Loss of trust’, ‘Power imbalance’ and ‘Engagement reduction’ all of which indicate a negative impact on the TR. Theme 2, ‘Nursing dilemma’, with the subtheme of ‘Dehumanisation’, highlights the conflicts faced by nurses with possible effects on the TR. Theme 3, ‘Reinforcement’, suggests improvements in the TR following coercive measures.

Dziopa and Ahern (2008) identify providing support, meeting at eye level, and empathy, among other things, as important characteristics of the TR, whereas Goodman et al. (2020) highlights trust as a crucial prerequisite. Two of the three developed themes, with subthemes, raise the question of whether it is possible for nurses involved in coercive measures to fulfil these important characteristics of the TR. These effects seem also to make it difficult for nurses to be therapeutically effective. A dysfunctional TR can also hinder efficient therapeutic treatment by affecting adherence. Tessier et al. (2017) found that traumatic experiences during psychiatric treatment are significantly associated with low adherence, which in turn supports the subtheme of ‘Engagement reduction’. This has implications not only for the TR but also for the healthcare system as a whole. Non‐adherence affects not only health outcomes but leads to readmissions and therefore increases healthcare costs enormously (Jayasree et al. 2024). Adherence, in turn, is linked to the TR (McCabe et al. 2012). This also consequently illustrates the ‘nursing dilemma’ found in the results of this integrative review.

With regard to the ‘Nursing dilemma’, it must be taken into account that moral distress among nurses results in depersonalisation, emotional exhaustion, cynicism, a poorer moral climate, a lower sense of coherence and a lower experience of moral support (Lamoureux et al. 2024). It is striking that only nurses described a positive effect of coercive measures on TR. It should be considered that such compliance from patients may stem from a fear of encountering additional coercive measures rather than reflecting a true enhancement of the TR. Berring et al. (2024) reports that patients adopt a facade of compliance with staff expectations as a coping strategy. It can also be seen as a resignation, which involves simply following the rules and complying with what they are told, to avoid trouble. Norvoll and Pedersen (2016) report that power imbalance caused by coercion‐induced leads patients to attempt resistance, escape, or avoid coercion by using various strategies of counter‐power. These strategies could be active, such as escape or vehement resistance. Alternatively, more passive approaches include avoiding services, feigning compliance, and ‘playing the game’ or actively taking responsibility to foster cooperative and trusting relationships.

Especially in cases of dysfunctional nursing behaviour, where nurses intentionally dehumanise themselves to protect from emotional harm, it is important to avoid unjustified and unprofessional use of coercive measures in the form of power imbalance.

Studies reporting on the experience of patients and nurses coercive measures frequently indicate negative emotions such as anger, fear and trauma (Chieze et al. 2019; Power et al. 2020). In this regard, nurses should be aware of the emotions they experience and not allow them to influence their decisions in connection with coercive measures. Research from Lerner et al. (2015) shows that emotions are powerful, pervasive and sometimes harmful or beneficial drivers of decision‐making.

Hartley et al. (2020) highlight a problematic issue in relation to developing and maintaining good TR. They state that there is little evidence that nurses are supported in this, despite it being recognised as a core element of psychiatric nursing and essential for positive patient outcomes. So, what can be done when the TR gets damaged? Based on findings from Berring et al. (2024), recovery from coercion is both a systemic and personal process influenced by the staff's ability to create an environment that supports continuous humane and empathic contact and promotes a sense of safety.

## 6 Strengths & Limitations

The strength of the research project lies in its integrative review methodology. This includes a thorough search in the major databases in both English and German and an in‐depth engagement with the content, carefully analysed by three reviewers. The result is a profound understanding of the topic. The use of the PRISMA checklist ensures transparency and traceability. In addition, the findings are relevant to practice, as they raise awareness of the fragility of the TR in the context of coercive measures. Potential publication bias includes the language restriction and the inclusion of only peer‐reviewed publications. One limitation is that although the studies identify a deconstructive effect of the TR, they do not provide detailed explanations for this. Despite differing views on coercion between patients and nurses, none of the included studies involved consumers or applied participatory methods such as codesign. Consequently, their perspectives are missing from the process of evidence generation. Another limitation is the subjective nature of the assessment of the TR, which may lead to inconsistencies in the results. Although the focus on the nurse–patient TR was crucial, given its central role in acute psychiatric care, a broader perspective including other professionals and aspects of the ward environment may provide additional insights.

## 7 Conclusion

This integrative review clearly shows that coercive measures have a destructive impact, ranging from mild to severe, on the TR between patients and nursing staff, leading to trust problems, power imbalances, and reduced engagement. Additionally, these measures result in nursing dilemmas and dehumanisation. Positive effects on the TR are rare and predominantly reported by nurses. The results show that when coercive measures harm the TR, they simultaneously compromise a fundamental aspect of psychiatric care. The lack of TR can encourage behaviours that result in further coercive measures. This can lead to a negative cycle that has detrimental effects for everyone involved. The stability of the TR therefore appears to be an important factor in reducing coercive measures. It is especially important for nurses responsible for the TR to be aware of the impact of coercive measures. To effectively reduce coercive measures, mental health systems need to adopt a human rights‐based approach. The United Nations (2017) advocates non‐coercive models, such as the soteria model, respite homes, and mental health crisis units as alternatives to the biomedical paradigm of psychiatric hospitalisation under coercion. Moreno‐Poyato et al. (2021) emphasise that a key measure of the TR is the absence of coercive measures.

### 7.1 Relevance for Further Research

Future research should actively involve people with lived experience and apply principles of codesign to ensure that findings reflect both clinical and consumer perspectives. Building on this, studies should also consider the longitudinal nature of coercive practices in order to better understand their impact on the TR. The following aspects should be considered: What was the TR like before the intervention, how did it take shape during the triggering event of the coercive measure, how did it develop during the implementation of the measure, what was it like after the coercive measure ended, and what is helpful to restore the TR? The subtheme of engagement reduction could evolve into a central focus for future research, as elements like loss of trust, power imbalance, and dehumanisation may also contribute to diminished involvement. Gaining deeper insight into these relationships is crucial for designing targeted strategies to enhance the TR and, consequently, patient participation. Over time, this may lead to better treatment outcomes and increased efficiency within the healthcare system.

### 7.2 Relevance for Clinical Practice

Nurses in acute psychiatric care need to be aware that coercive measures have a negative impact on the TR. Furthermore, it is important for nurses, who are responsible for the TR, to be conscious of the negative cycle in order to counteract anti‐therapeutic behaviour. Additionally, nursing staff should critically question coercive measures and develop sustainable patient‐centred, recovery‐oriented alternatives to strengthen trust and cooperation between patients and nursing staff and achieve more effective treatment outcomes in the long‐term. According to Berring et al. (2024), a supportive environment is needed during and after coercive measures, where patients can engage in communicative exchanges with nurses. This environment should convey a sense of dignity, respect, and safety. Experiencing dignity and respect is central to the recovery process. Patients maintain their dignity and feel respected when they are acknowledged as individuals. As noted by Ling et al. (2015), structured debriefings can help process coercive measures, restore trust, and facilitate future treatment plans. This requires management support by creating the necessary conditions to support efforts to create and maintain a stable TR. The establishment and maintenance of a stable TR must play a central role in psychiatric care, as all of its characteristics are an important protective factor against dehumanisation and the use of coercive measures.

## Ethics Statement

The authors have nothing to report.

## Conflicts of Interest

The authors declare no conflicts of interest.

## Data Availability

The authors have nothing to report.
